# LITE-1 mediates behavioral responses to X-rays in *Caenorhabditis elegans*

**DOI:** 10.3389/fnins.2023.1210138

**Published:** 2023-08-10

**Authors:** Kelli E. Cannon, Meenakshi Ranasinghe, Paul W. Millhouse, Ayona Roychowdhury, Lynn E. Dobrunz, Stephen H. Foulger, David M. Gauntt, Jeffrey N. Anker, Mark Bolding

**Affiliations:** ^1^Department of Vision Sciences, School of Optometry, University of Alabama at Birmingham, Birmingham, AL, United States; ^2^Department of Neurobiology, Heersink School of Medicine, University of Alabama at Birmingham, Birmingham, AL, United States; ^3^Department of Radiology, Heersink School of Medicine, University of Alabama at Birmingham, Birmingham, AL, United States; ^4^Department of Chemistry, Clemson University, Clemson, SC, United States; ^5^Department of Materials Science and Engineering, College of Engineering, Computing and Applied Sciences, Clemson University, Clemson, SC, United States

**Keywords:** noninvasive neuromodulation, X-rays, X-genetics, optogenetics, lite-1, X-ray optogenetics

## Abstract

Rapid sensory detection of X-ray stimulation has been documented across a wide variety of species, but few studies have explored the underlying molecular mechanisms. Here we report the discovery of an acute behavioral avoidance response in wild type *Caenorhabditis elegans* to X-ray stimulation. The endogenous *C. elegans* UV-photoreceptor protein LITE-1 was found to mediate the locomotory avoidance response. Transgenic expression of LITE-1 in *C. elegans* muscle cells resulted in paralysis and egg ejection responses to X-ray stimulation, demonstrating that ectopic expression of LITE-1 can confer X-ray sensitivity to otherwise X-ray insensitive cells. This work represents the first demonstration of rapid X-ray based genetically targeted (X-genetic) manipulation of cellular electrical activity in intact behaving animals. Our findings suggest that LITE-1 has strong potential for use in this minimally invasive form of neuromodulation to transduce transcranial X-ray signals for precise manipulation of neural activity in mammals, bypassing the need for invasive surgical implants to deliver stimulation.

## 1. Significance statement

Here we report the discovery of LITE-1 dependent behavioral responses to X-radiation. Importantly, we show that LITE-1 can confer X-ray sensitivity when transgenically expressed in otherwise X-ray insensitive cells. This is the first demonstration of acute X-ray mediated modulation of cellular electrical activity that has similar functionality to optogenetics, but avoids the need for surgical implantation of optic fibers and improves targeting capabilities.

## 2. Introduction

Neuroscientists today can manipulate neural activity with unprecedented precision. Spatially restricted genetically targeted cell-type-specific expression of proteins gives researchers the ability to test very specific hypotheses about nervous system function. Optogenetic techniques employ light to modulate neuronal activity using light-sensitive photoreceptor proteins (Boyden et al., 2005). Since its debut in 2005, optogenetics has led to many new discoveries, been extensively developed and adapted, and become an indispensable tool in the neuroscientist’s armamentarium (Josselyn, 2018). Applying optogenetics *in vivo*, however, is hindered by optical absorption and scattering in thick tissue. To guide light through the skull to specific brain regions in mammalian models, invasive cranial windows or optical fiber implants are required (Aravanis et al., 2007). Moreover, these light guides have limited fields of view and cannot be easily moved to new regions. To avoid these limitations, researchers have been investigating the use of alternative wavelengths, such as near-infrared (NIR) and X-rays, which are capable of efficiently transmitting externally generated control signals through the skull and brain tissue, allowing for cleaner experiments with results that are less confounded by technical shortcomings.

Researchers have been studying how X-rays affect cells and organisms since X-rays were discovered in 1895 and have reported X-ray responses in retinal photoreceptors (Baily and Noell, 1958; Bachofer and Wittry, 1961; Hunt and Kimeldorf, 1962; Garcia and Buchwald, 1963; Doly et al., 1980), cellular processes (especially related to cell replication and death, Barron and Seki, 1952; Puck and Marcus, 1956; Harmon and Allan, 1988; Boothman et al., 1994; Maier et al., 2015), and animal behavior (Smith et al., 1963; Dedrick and Kimeldorf, 1974; Kernek and Kimeldorf, 1975). Recently several groups have begun investigating a technique known as X-ray optogenetics for minimally invasive manipulation of neural activity in deep brain areas (Berry et al., 2015; Bartley et al., 2019; Chen et al., 2021; Matsubara et al., 2021). The approach uses X-rays to excite radioluminescent particles (RLPs) delivered to the extracellular space surrounding neurons; these RLPs convert the incident X-ray energy into visible light, which in turn activates transgenically expressed photoreceptors. The main difficulty with X-ray optogenetics is producing enough light to generate a meaningful change in neural activity using reasonable X-ray doses and RLP concentrations. Use of a highly sensitive receptor protein that is capable of generating a larger current from fewer photons would facilitate the approach. We therefore searched the literature for more sensitive photoreceptor proteins, including G-protein coupled receptors (GPCRs), which benefit from multiple amplification steps such that a single photon absorbed by the GPCR results in the opening of many channels.

We came across LITE-1, an unusual UV-sensitive photoreceptor protein found in *C. elegans* that has been found to absorb photons orders of magnitude more efficiently than several common opsins (Gong et al., 2016). Unrelated to other known photoreceptor families, LITE-1 belongs to a family of 7-transmembrane-domain invertebrate gustatory receptors and the exact nature of LITE-1’s photosensitivity is still an area of active research. G-proteins have been found to act downstream of LITE-1 activation in ASJ neurons (Liu et al., 2010), but the receptor’s inverted membrane topology and lack of homology with other GPCRs (Gong et al., 2016) suggest that it may not be considered a true GPCR. LITE-1 mediates locomotory avoidance behavior in wild type *C. elegans* in response to UV (Edwards et al., 2008). We were surprised to discover that X-rays alone elicit a behavioral avoidance response in wild type *C. elegans* in the absence of RLPs. The response was absent in LITE-1 deficient worms, suggesting that LITE-1 plays a critical role in mediating X-ray avoidance behavior. Importantly, we show that LITE-1 can confer X-ray sensitivity when transgenically expressed in otherwise X-ray insensitive cells. This is the first demonstration of X-ray mediated modulation of cellular electrical activity that has similar functionality to optogenetics, while avoiding the disadvantages associated with the use of visible light stimulation.

## 3. Methods

### 3.1. X-ray avoidance experiments

#### 3.1.1. Experimental model

*C. elegans* roundworms were maintained at 20°C on nematode growth medium (NGM) agar plates seeded with OP50 *E. coli* lawns (Brenner, 1974). A wild type strain (N2, Bristol) was assayed, along with three mutant strains with severely defective responses to UV light due to mutations *lite-1*(*ce314*) and/or *gur-3*(*ok2245*). These strains of *C. elegans* were obtained from the Caenorhabditis Genetics Center, which is funded by the NIH Office of Research Infrastructure Programs (P40 OD010440).

#### 3.1.2. X-ray stimulation

X-rays were generated by a iMOXS-MFR W-target X-ray unit with polycapillary optics (XOS) to focus the X-ray beam to a FWHM diameter of 0.85 mm at the level of the agar surface, located approximately 5 cm from the tip of the capillary attachment. The unit was operated at 50 kV and the current was varied from 0 to 600 μA to achieve different stimulation intensities. At the highest intensity, a radiation dose of 1 Gy/s was approximated using RADSticker dosimeter stickers (JP Laboratories, Inc.). The dose decreased to approximately 0.2 Gy/s when the X-ray unit was operated at 25% of the maximum current, i.e., 150 μA. Although the exact X-ray doses may vary somewhat from these values, we expect these dose estimates to be accurate within a factor of two. An internal X-ray shutter was used to control the timing of the X-ray stimulation.

#### 3.1.3. Imaging setup

**Figure 1:**
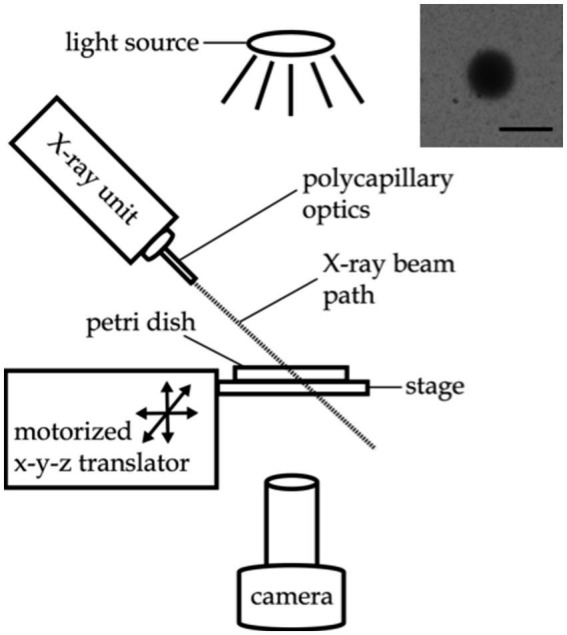
Experimental setup for X-ray avoidance and egg ejection experiments. Inset image: X-ray beam visualized with a piece of radiochromic film placed on the agar surface. Scale bar indicates 1 mm.

Worm behavioral responses to X-ray stimulation were recorded on a custom imaging setup, as shown in Figure 1. Video data was recorded using an Amscope MU1003 10MP CMOS camera with a 0.7X-180X magnification lens. Back lighting was provided by a white LED source with a diffuser. The X-ray unit was mounted above the stage. The stage was positioned using a motorized x-y translator controlled by the joystick of an Xbox controller (Microsoft, Redmond WA). The entire imaging setup was enclosed in a steel box with no detectable external X-ray leakage during use.

The irradiation zone—a sharply defined, 0.85 mm diameter spot on the agar surface—was visualized using a small piece of radiochromic film (Gafchromic, XR-QA2) placed on the agar surface (see Figure 1 inset). The camera recording software (Amscope 3.7) was used to annotate the video monitor with a circle outlining the irradiation zone as visualized with the radiochromic film for targeting purposes.

X-ray shutter timing was synced to video timing *post hoc* using a second video recording device outside of the steel enclosure that acquired audio data. The camcorder collected video of the live recording feed with a timestamp displayed as well as audio data capturing the click of the X-ray shutter opening and closing. The timestamp on the video recording updated at a rate of 1 Hz, limiting the precision of the timing data to ±1 s.

#### 3.1.4. Experimental design

All experiments were conducted on OP50-lawned 100 mm NGM plates inoculated with approximately 20–50 worms 24–48 h prior. Looking at one worm per trial, a total of 130 trials were conducted on worms from 11 different plates, targeting 3 to 20 individual worms from each plate. A small piece of radiochromic film was placed on the agar surface of each plate before the plate was placed, uncovered, on the imaging stage in the irradiation chamber. After calibrating the location of the X-ray spot with the radiochromic film as described above, an adult hermaphrodite was selected and the stage was moved to position the worm in the center of the calibrated X-ray spot. Each trial began with 30 s during which baseline behavior was recorded with the X-ray shutter closed. After 30 s, if the worm moved out of the center of the calibrated spot, the stage was moved to reposition the worm in the irradiation zone. Once the worm was in position, the shutter was opened to deliver a 10 s X-ray pulse. The pulse was terminated by closing the shutter, and worm behavior was recorded for another 10 s after stimulation offset.

For the dose-rate response experiments, behavioral responses of wild type worms to increasing intensities of X-ray stimulation were recorded. Five X-ray intensities between zero and the maximum intensity deliverable by our iMOX X-ray unit were tested. Intensities of approximately 0, 0.2, 0.5, 0.7, and 1.0 Gy/s were achieved by setting the current applied to the X-ray tube to 0, 150, 300, 450, and 600 μA, respectively. Twenty trials of each the null and highest intensities, and 10 trials at each of the three intermediate intensities were collected, for a total of 70 trials. The five intensities were delivered in an interspersed and randomized order.

For the strain comparison experiments, the responses of three mutant strains of *C. elegans* were tested at the null and maximum X-ray intensities, as described above. Ten trials at each of the two intensities were conducted for each strain for a total of 60 trials.

#### 3.1.5. Data analysis

Video recordings were stabilized in Matlab (due to plate movement from recentering the target worm in the irradiation zone) and analyzed using WormLab software (MBF Bioscience, version 2019.1.1) to track and measure the activity levels of worm subjects. Prior analysis of an independent preliminary dataset (unpublished data) found the behavioral metric most sensitive to the X-ray avoidance response was *activity*, in units of body area per minute, as defined by CeleST tracking software (Restif et al., 2014). Activity time courses were calculated in WormLab and combined with stimulation timing info in Matlab (R2019a) to calculate the peak change in activity—the maximum activity reached during the 10 s X-ray pulse minus the average activity during 30 s baseline period—normalized to the baseline activity for each trial. We defined a positive avoidance response as an increase in activity greater than 45%, using the largest increase observed in the negative control condition to guide threshold selection. Three trials were excluded due to tracking problems. The three images in Figures 3D–F were created by calculating the difference between the maximum and minimum intensities of each pixel across all frames during the 10 s X-ray pulse and subtracting the difference image from the green component of the RGB image of the last frame. For statistical analysis of the X-ray intensity response plot in Figure 3J, a Kruskal-Wallace test was performed, followed by a series of one-sided unpaired Mann Whitney U tests to compare each of the four non-sham stimulation conditions to the sham condition. For statistical analysis of the X-ray avoidance strain comparison data in Figure 4E, a Scheirer-Ray-Hare test was performed, followed by a series of one-tailed unpaired Mann Whitney *U* tests to compare the responses to sham versus X-ray stimulation for each strain. Bonferroni correction was used to adjust Mann Whitney *p*-values for multiple comparisons.

**Figure 2:**
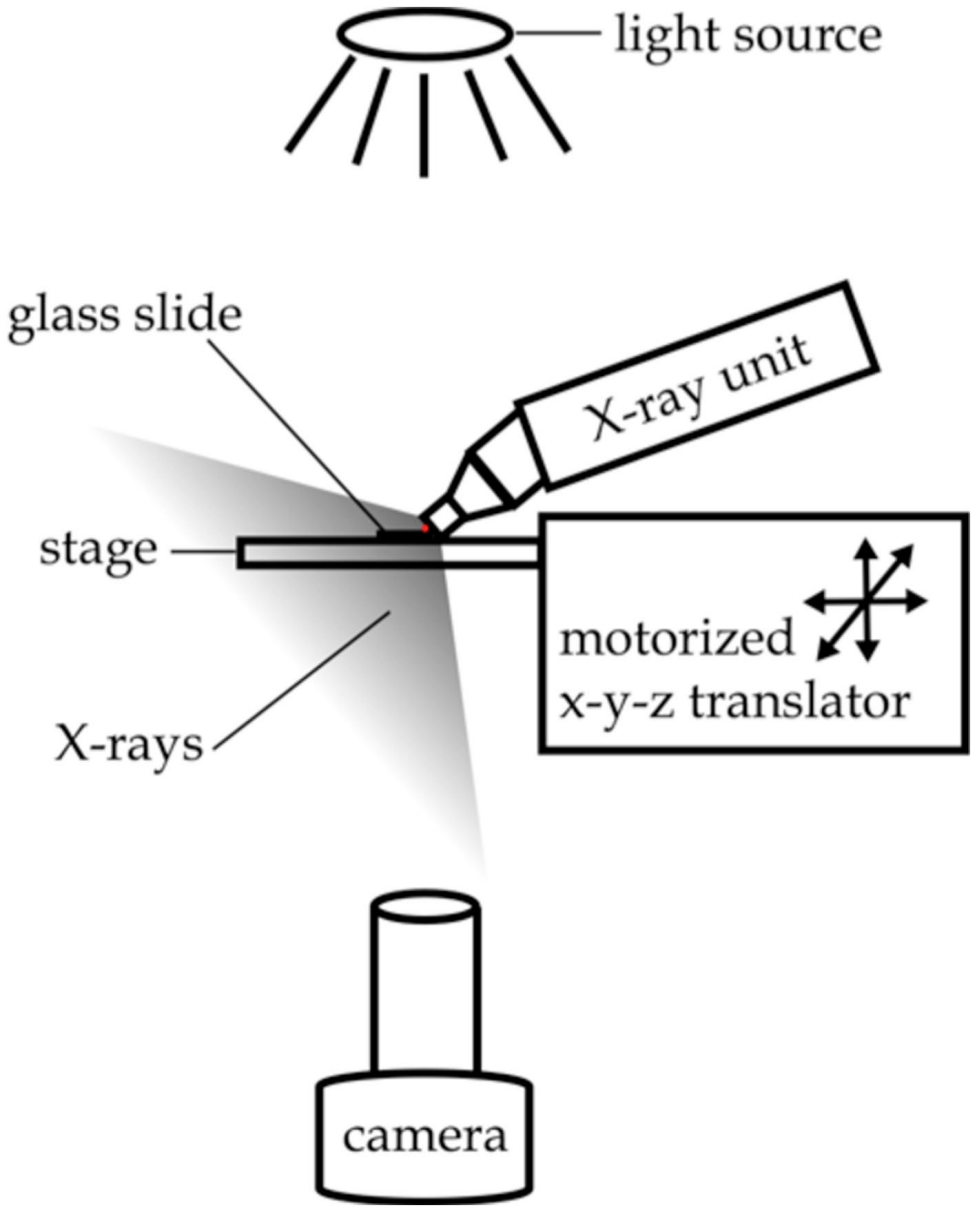
Experimental setup for X-ray paralysis experiments. The red dot indicates the location of the nematode.

**Figure 3:**
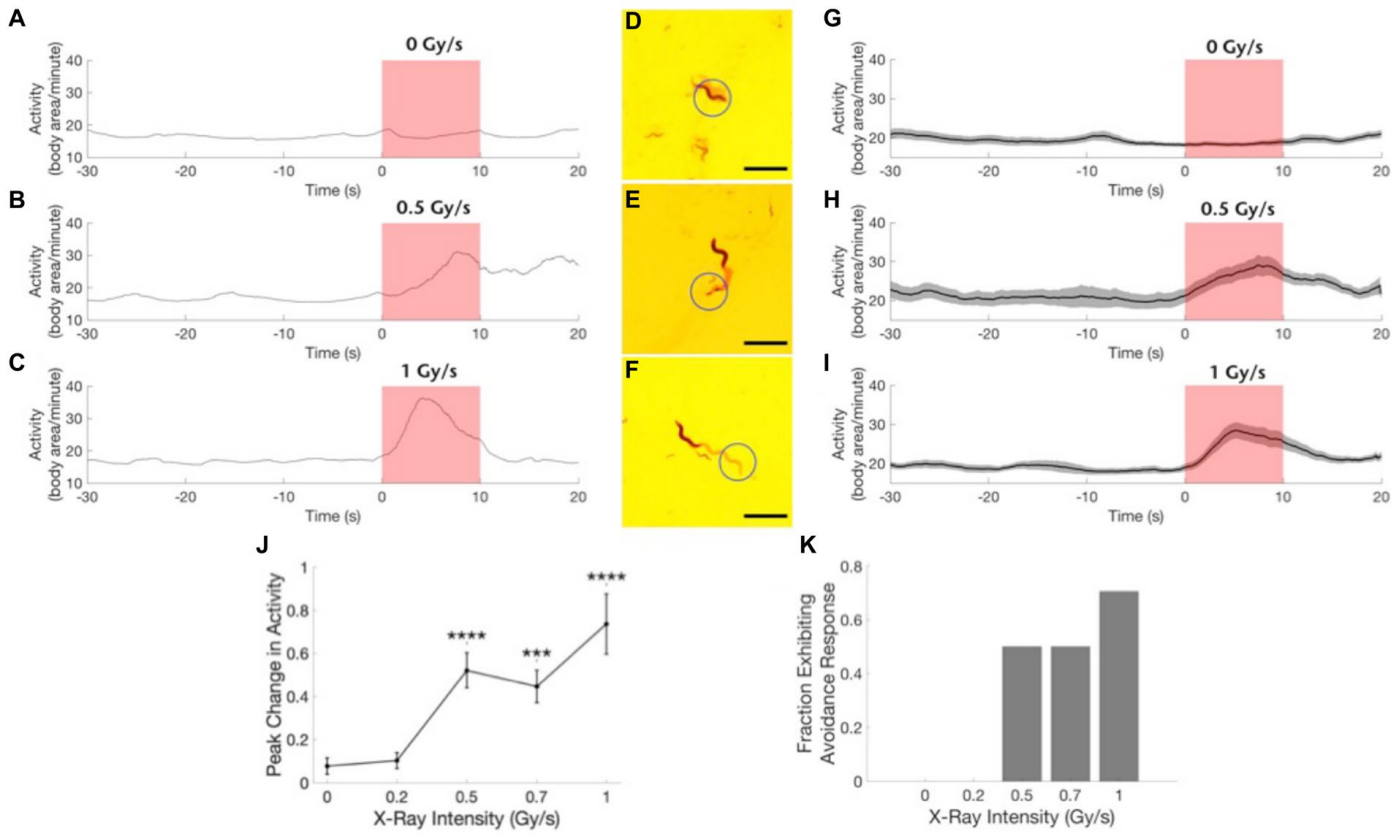
Wild type worms exhibit an avoidance response to focused X-ray stimulation. **(A–C)** Example traces of worm activity before, during, and after X-ray stimulation at dose rates of 0 Gy/s **(A)**, 0.5 Gy/s **(B)**, and 1 Gy/s **(C)**. Red shading indicates the timing of the X-ray pulse. **(D–F)** Images showing worm locomotion over the course of the 10-s X-ray pulse. Circle indicates the location of the X-ray beam. The light red paths show the area covered by the worm during the pulse. Worm is shown in its position at the end of the pulse. Scale bars indicate 1 mm. **(G–I)** Activity time courses averaged across worms before, during, and after X-ray stimulation at dose rates of 0 Gy/s **(G)**, 0.5 Gy/s **(H)** and 1 Gy/s **(I)**. Gray shading indicates the standard error of the mean time series. Red shading indicates the timing of the X-ray pulse. *N* = 20 for 0 Gy/s condition. *N* = 10 for 0.5 Gy/s condition. *N* = 17 for 1 Gy/s condition. **(J)** The peak change in activity during the X-ray pulse as a fraction of mean baseline activity is shown as a function of stimulation intensity. ***Bonferroni corrected *p* < 0.001, ****Bonferroni corrected *p* < 0.0001. **(K)** The fraction of worms exhibiting an avoidance response, defined as *a* > 45% increase in activity, is shown for each stimulation intensity.

**Figure 4:**
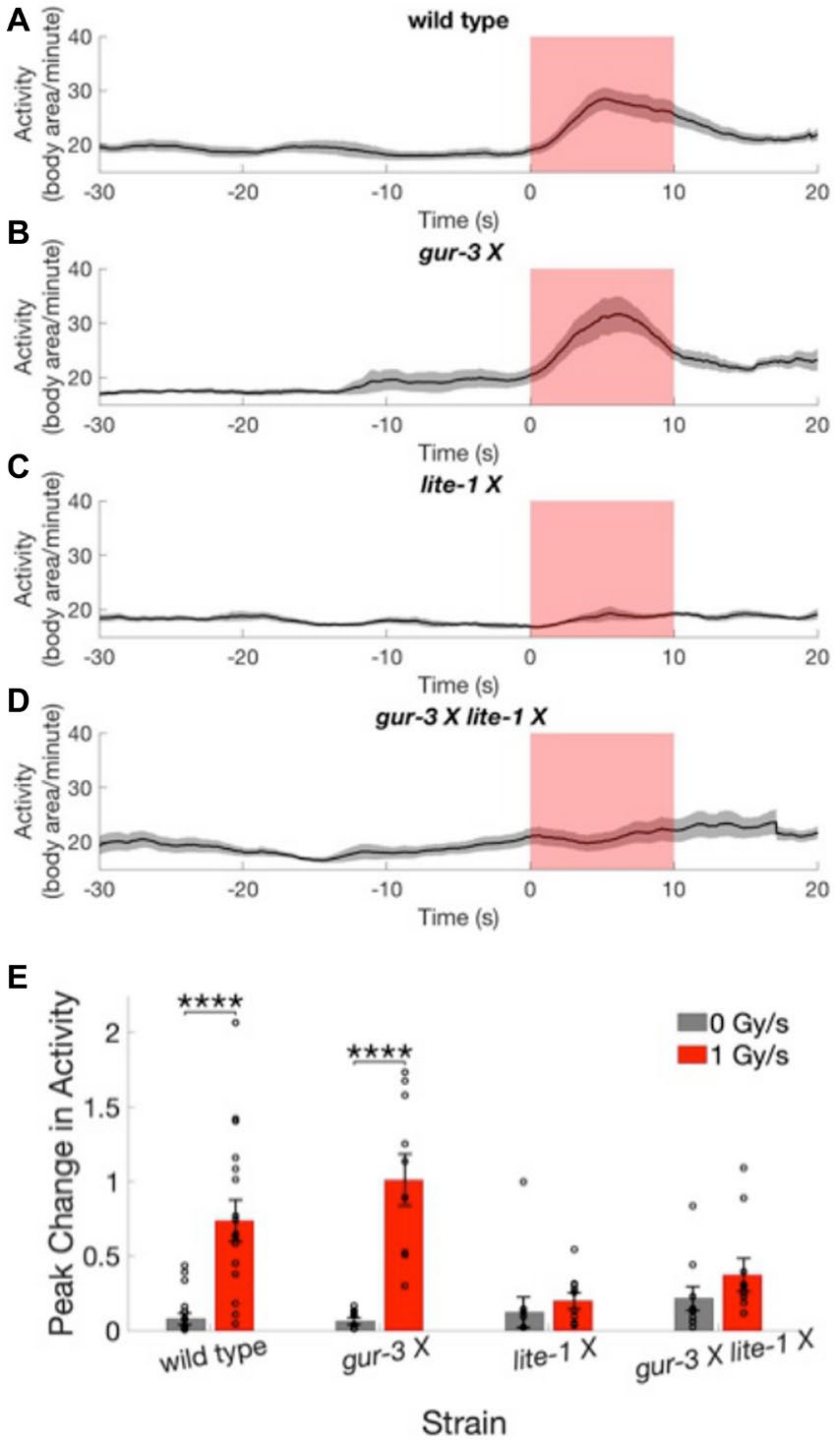
Worms lacking functional LITE-1 have severely defective X-ray avoidance responses. **(A–D)** Activity time courses averaged across worms of the wild type strain **(A)** and 3 mutant strains **(B–D)** before, during, and after X-ray stimulation at a dose rate of 1 Gy/s. Gray shading indicates the standard error of the mean time series. Red shading indicates the timing of the X-ray pulse. *N* = 20 for wild type strain. *N* = 10 for mutant strains. **(E)** The peak change in activity during the X-ray pulse as a fraction of mean baseline activity is shown for the four strains at 0 and 1 Gy/s stimulation intensities. Error bars indicate standard error. ****p < 0.0001.

### 3.2. X-ray paralysis experiments

#### 3.2.1. Experimental model

*C. elegans* roundworms were maintained at 20°C on nematode growth medium (NGM) agar plates seeded with OP50 *E. coli* lawns (Brenner, 1974). The wild type N2 strain was used as a negative control. N2 nematodes were obtained from the Caenorhabditis Genetics Center, which is funded by the NIH Office of Research Infrastructure Programs (P40 OD010440). *Xuls98 [pmyo-3::lite-1::1D4::SL2::YFP]* worms (henceforth referred to as *pmyo-3::lite-1* worms) were generously provided by the lab of Dr. Shawn Xu. This transgenic strain that expresses *lite-1* in muscle cells using a *myo-3* promoter has been shown to respond to UV with muscle contraction leading to paralysis and sometimes egg ejection (Gong et al., 2016).

#### 3.2.2. X-ray stimulation

X-rays were generated by a portable Amptek Mini-X X-ray unit (Ag target, nozzle and filters removed). In contrast to the focused X-ray beam produced by the iMOXS unit for the X-ray avoidance experiments, the Mini-X unit produced a 120° cone of X-rays, resulting in relatively uniform diffuse irradiation over the entire field of view. The unit was operated at 20 kV and the current was varied from 0 to 198 μA to achieve different stimulation intensities. RadCal 9010 X-ray dosimeter with a RadCal10x6 ionization chamber was used to measure tube output. At the worms’ location approximately 1 cm from the X-ray focal spot, dose rates of 0, 0.19, 0.38, 0.56, and 0.74 Gy/s were calculated for the five X-ray intensity settings employed—i.e., when 0, 50, 100, 150, and 198 μA currents, respectively, were applied to the X-ray tube. The worms were in a 5 μL drop of M9 buffer that had a maximum height of approximately 0.5 mm. Assuming an average photon energy of 10 keV, 0.5 mm of water would attenuate about 1 out of every 100 photons administered. A custom-built X-ray shutter was used to control the timing of the X-ray stimulation.

#### 3.2.3. Imaging Setup

Worm behavioral responses to X-ray stimulation were recorded on a custom imaging setup, as shown in Figure 2. Video data was recorded using an Amscope MU1003 10MP CMOS camera with a 0.7X-180X magnification lens. Back lighting was provided by a white LED source with a diffuser. The X-ray unit was mounted above the stage, and the stage was positioned using manual x-y-z translators. A custom-built triggering device controlled the video recording software and the X-ray shutter, automating the recording and stimulation processes.

#### 3.2.4. Experimental design

For each trial a fresh adult hermaphrodite was placed in a 5 μL drop of M9 buffer on a glass slide with no coverslip, and the slide was positioned on the stage touching the nozzle of the X-ray unit as shown in Figure 2. Video recording of the animals’ swimming behavior began after a 60 s recovery period. Twenty seconds of baseline behavioral data was collected prior to a 20 s pulse of X-ray stimulation. Behavior was recorded for another 20 s after the termination of the X-ray stimulation. Importantly, the unfocused X-ray stimulation provided by the Mini-X unit made it such that the worm was not able to move out of the irradiation zone to escape the stimulation, as was the case for the avoidance experiments.

For the dose–response experiments, behavioral responses of *pmyo-3::lite-1* worms to increasing intensities of X-ray stimulation were recorded. Five X-ray intensities between zero and the maximum intensity deliverable by our Mini-X X-ray unit were tested. Intensities of 0, 0.19, 0.37, 0.56, and 0.74 Gy/s were achieved by setting the current applied to the X-ray tube to 0, 50, 100, 150, and 198 μA, respectively. Since the worms were not able to escape the diffuse, unfocused X-ray stimulation as was the case with the X-ray avoidance experiments, all worms were exposed to X-radiation for the entire duration of the 20 s pulse. As a result, a given X-ray intensity deposited approximately the same X-ray dose in each worm across trials. Ten trials were collected for each dose condition, resulting in a total of 50 trials. The five X-ray doses were delivered in an interspersed and randomized order.

For the strain comparison experiments, the behavioral responses of wild type and *pmyo-3::lite-1* nematodes to the null and maximum X-ray doses were recorded. Twelve trials at each of the two intensities were conducted for each strain for a total of 48 trials.

#### 3.2.5. Data analysis

**Figure 5:**
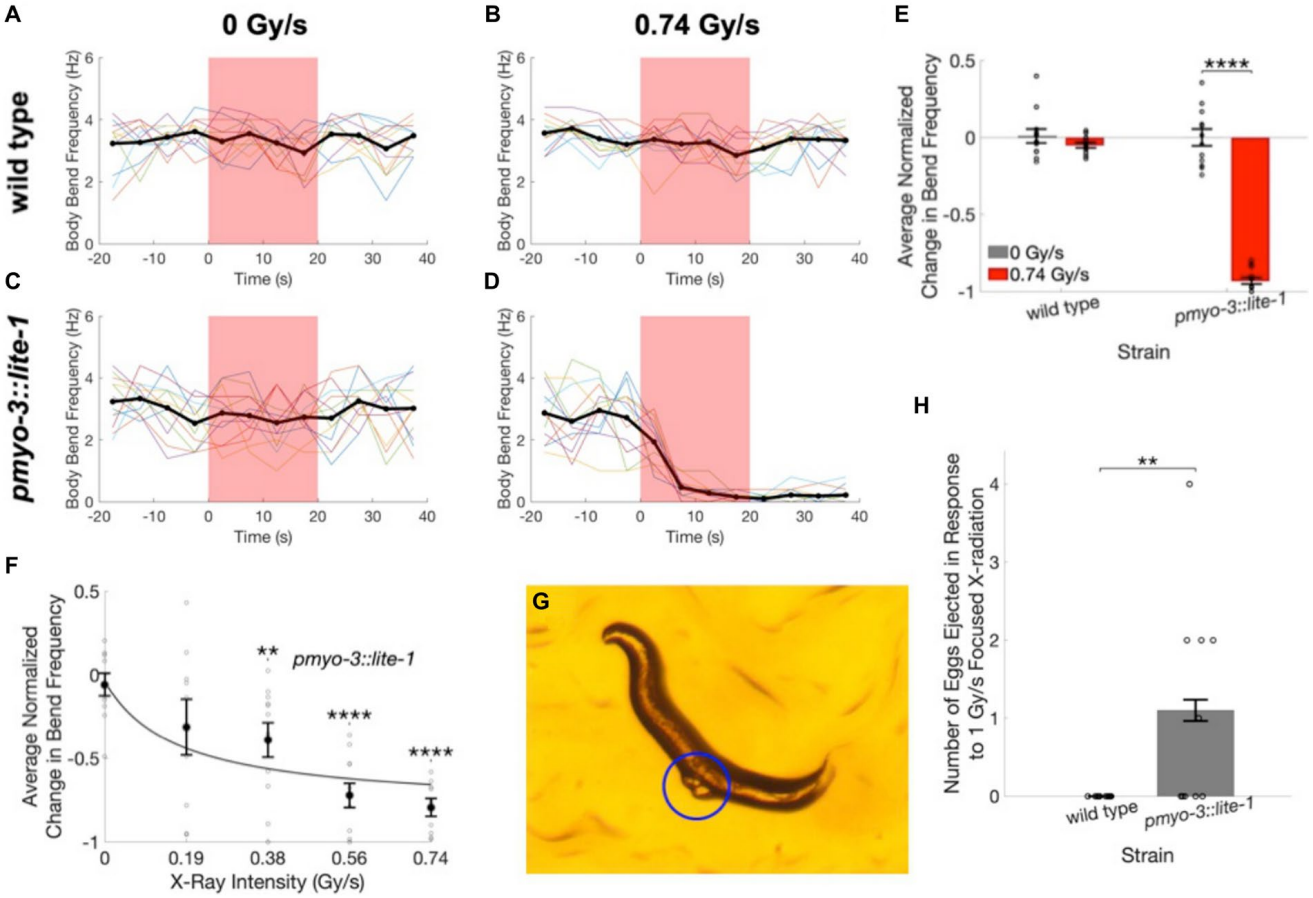
Worms expressing LITE-1 in muscle cells exhibit a paralysis response to X-ray stimulation. **(A–D)** Time courses of wild type **(A,B)** and *pmyo-3::lite-1* **(C,D)** *C. elegans* activity quantified as the number of body bends per 5-s interval before, during, and after unfocused X-ray stimulation at dose rates of 0 Gy/s **(A,C)** and 0.74 Gy/s **(B,D)**. Thin colored traces represent individual worms, and the mean across worms is indicated by the thick black line. Red shading indicates the timing of the X-ray pulse. N = 12 for each strain and stimulation condition. **(E)** The average change in body bend frequency after the X-ray pulse as a fraction of mean baseline bend frequency is shown for wild type and *pmyo-3::lite-1* worms at 0 and 0.74 Gy/s stimulation intensities. Error bars indicate standard error. ****p < 0.0001. **(F)** The average change in body bend frequency of *pmyo-3::lite-1* worms is shown as a function of stimulation intensity. Values for individual worms are indicated by circles. *N* = 10 at 0 Gy/s. *N* = 9 at 0.19 and 0.74 Gy/s. N = 11 at 0.38 and 0.56 Gy/s. Error bars indicate standard error. **Bonferroni corrected *p* < 0.01, ****Bonferroni corrected *p* < 0.0001. **(G)** Image of a *pmyo-3::lite-1* worm that ejected two eggs, indicated by blue circle, when exposed to 1 Gy/s focused X-ray stimulation. **(H)** The number of eggs ejected by wild type and *pmyo-3::lite-1* nematodes in response to a 15 s pulse of 1 Gy/s focused X-ray stimulation. Error bars indicate standard error. Values for individual worms are indicated by circles. **p < 0.01.

*C. elegans* activity was quantified by manually counting the number of body bends in each 5 s video segment. The change in body bend frequency was calculated as the average bend frequency during the 20 s after X-ray stimulation minus the average bend frequency during the 20 s baseline period. This value was divided by the worm’s baseline bend frequency in order to normalize the metric with respect to the worm’s baseline activity level. A Scheirer-Ray-Hare test followed by post-hoc unpaired one-sided Mann Whitney U tests were used to determine the significance of the changes in body bend frequency observed in response to 0.74 Gy/s X-ray stimulation compared to those observed for the sham stimulation condition for each of the strains in Figure 5E. For statistical analysis of the X-ray dose rate response plot in Figure 5F, a Kruskal-Wallace test was performed, followed by a series of unpaired one-tailed Mann Whitney U tests to compare each of the four non-sham stimulation conditions to the sham condition. Bonferroni correction was used to adjust Mann Whitney *p*-values for multiple comparisons.

### 3.3. Egg ejection experiments

#### 3.3.1. Experimental model

*C. elegans* roundworms were maintained at 22°C on nematode growth medium (NGM) agar plates seeded with OP50 *E. coli* lawns (Brenner, 1974). The wild type N2 strain was used as a negative control. N2 nematodes were obtained from the Caehabditiris Genetics Center, which is funded by the NIH Office of Research Infrastructure Programs (P40 OD010440). *Pmyo-3::lite-1* worms were generously provided by the lab of Dr. Shawn Xu.

#### 3.3.2. X-ray stimulation

X-ray stimulation for the egg ejection experiments was delivered using the iMOXS-MFR focused X-ray unit and setup described above for the avoidance experiments. Two dose rates, 0 and 1 Gy/s, were tested by applying 0 and 600 µA currents, respectively, to the X-ray tube at a voltage of 50 kV.

#### 3.3.3. Imaging setup

The imaging setup was as described above for the avoidance experiments.

#### 3.3.4. Experimental design

All experiments were conducted on OP50-lawned 100 mm NGM plates inoculated with approximately 20–50 worms 72 h prior. Looking at one worm per trial, a total of 40 trials were conducted on worms from 8 different plates, targeting 3 to 7 individual worms from each plate. As described above for the avoidance experiments, a small piece of radiochromic film was used to calibrate the location of the X-ray beam on the agar surface. An adult hermaphrodite was selected and the stage was moved to position the worm in the center of the calibrated X-ray spot. Each trial began with 10 s during which baseline behavior was recorded with the X-ray shutter closed. Then the shutter was opened to deliver a 15 s X-ray pulse.

The responses of wild type and *pmyo-3::lite-1* transgenic nematodes were tested at the null and maximum X-ray intensities, as described above. Ten trials at each of the two intensities were conducted for each strain for a total of 40 trials.

#### 3.3.5. Data analysis

Egg ejections during the X-ray pulse were manually tallied for each worm. A one-sided unpaired Mann Whitney U test was used to compare the mean number of eggs ejected by wild type versus *pmyo-3::lite-1* nematodes in response to 1 Gy/s X-ray stimulation.

## 4. Results

### 4.1. Wild type *C. elegans* exhibit locomotory avoidance behavior in response to focused X-ray stimulation

To demonstrate the sensitivity of wild type *C. elegans* to X-rays, individual worms were positioned in the path of a focused X-ray beam that produced an irradiation area with a 0.85 mm full-width half maximum diameter at the level of the agar surface (Figure 1, inset). Wild type worms exhibited a robust increase in activity in response to 1 Gy/s X-ray stimulation (Figure 3; Supplementary Video 1). Figures 3A–C show example traces of the activity of wild type worms before, during, and after a 10 s pulse of focused X-ray stimulation at dose rates of 0 Gy/s (sham, 3A), 0.5 Gy/s (3B), and 1 Gy/s (3C). Figures 3D–F show images indicating nematode movement during the 10 s X-ray pulse. The nematodes are shown at their location at the termination of the pulse, and the red shading indicates the nematode’s trail, or the area covered by the nematode during the pulse. Figures 3C,F illustrate typical positive avoidance responses, wherein the worm increases forward locomotion to escape the irradiation zone, which is indicated by the blue circle. In contrast, there was no avoidance response to application of a sham stimulation (0 Gy/s, Figures 3A,D), and the worm remained within the circle for the duration of the sham stimulation. Additionally, when more than one worm was in view, only the worm exposed to the focused X-ray beam responded consistently. Figures 3G–I show activity traces averaged across worms for the three dose rates. Similar to the locomotory avoidance response seen in wild type worms exposed to UV stimulation (Edwards et al., 2008), the increase in activity manifested primarily as an increase in forward locomotion and sometimes involved reversals and omega bends. Nematode velocity increased during the first 5 s, with a significant response often observed within the first 2 s (example trace in Figure 3C, group average data in Figure 3I). After 5 s, the response dwindled as the nematode escaped the beam of radiation. At lower X-ray intensities, (i.e., 0.5 and 0.7 Gy/s), only 50% of worms exhibited a significant response (Figure 3K, defined as a > 45% increase in activity), response amplitudes were smaller (Figure 3J), and escape latencies were longer (not quantified, but seen as a later peak in activity in the time courses shown in Figures 3B,H compared to Figures 3C,I). Worm activity appeared unaltered by X-ray stimulation at either 0.2 Gy/s or the null intensity condition (Figures 3A,D,G,J,K). X-ray intensity, or dose rate, was found to significantly modulate the observed change in activity (Figures 3J,H(5) = 32.5, *p* = 1.5×10^−6^). Post-hoc pairwise comparisons revealed significant differences between the sham condition (0 Gy/s) and the 0.5 Gy/s (*U* = 11, Bonferroni corrected *p* = 2.6×10^−5^), 0.7 Gy/s (*U* = 20, Bonferroni corrected *p* = 8.1×10^−5^), and 1 Gy/s (*U* = 42, Bonferroni corrected *p* = 1.7×10^−5^) conditions.

### 4.2. Worms lacking functional LITE-1 have a dysfunctional X-ray avoidance response

We next tested whether either of 2 *C. elegans* photoreceptor proteins, LITE-1 or GUR-3, plays a role in the avoidance response to X-rays. Three mutant strains of *C. elegans* with mutated, dysfunctional *lite-1* [*lite-1* (*ce314*) *X*], *gur-3* [*gur-3* (*ok2245*) *X*] or both *lite-1* and *gur-3* [*lite-1* (*ce314*) *X gur-3* (*ok2245*) *X*] were subjected to sham and 1 Gy/s X-ray stimulation. Activity levels of the *gur-3* (*ok2245*) *X* mutant showed a significant increase in response to X-ray stimulation (Figure 4B; Supplementary Video 2), similarly to the wild type strain (Figure 4A). On average, the magnitudes of the increases were 74 ± 14% and 101 ± 17% of baseline activity for wild type and *gur-3* (*ok2245*) *X* strains, respectively, compared in negligible increases of 8 ± 4% and 6 ± 2% observed for the respective strains in response to sham stimulation [wild type *U* = 42, Bonferroni corrected *p* = 2.6×10^−5^; *gur-3* (*ok2245*) *X U* = 0, Bonferroni corrected *p* = 2.2×10^−5^; Figure 4E]. In contrast, the activities of the two strains with dysfunctional LITE-1—i.e., the *lite-1* (*ce314*) *X* mutant and the *gur-3* (*ok2245*) *X lite-1* (*ce314*) *X* double mutant—appeared largely unaltered by stimulation (Figures 4C–E, Supplementary Video 3, 4), suggesting that functional LITE-1 is required for the X-ray avoidance response.

### 4.3. LITE-1 confers X-ray sensitivity to muscle cells resulting in a paralysis response to X-ray stimulation

To determine whether transgenic expression of LITE-1 is capable of conferring X-ray sensitivity to otherwise X-ray insensitive cells, we looked at X-ray induced behavioral responses in *pmyo-3::lite-1* nematodes, which transgenically express LITE-1 in muscle cells. UV stimulation has been shown to result in LITE-1 mediated muscle contraction in this strain, leading to paralysis and egg ejection (Edwards et al., 2008; Bhatla et al., 2015; Gong et al., 2016). Using a swimming assay, we found that the *pmyo-3::lite-1* worms exhibited a robust paralysis response to diffuse, unfocused X-ray stimulation (Figures 5D,E; Supplementary Video 5) that was not observed in wild type nematodes (Figures 5B,E Supplementary Video 6). The average body bend frequency was found to decrease by 72 ± 5% in response to 0.74 Gy/s X-ray stimulation in the *pmyo-3::lite-1* strain (Figures 5D,E, *U* = 144, *p* = 1.8×10^−5^). Only negligible changes in bend frequency were observed at the same dose in the wild type strain (3 ± 2%, Figures 5B,E) and at the null X-ray dose in the *pmyo-3::lite-1* (2 ± 18%, Figures 5C,E) and wild type (0 ± 3%, Figures 5A,E) strains.

To determine whether the X-ray paralysis response in the *pmyo-3::lite-1* strain exhibits a typical dose–response relationship, we varied the current applied to the X-ray tube to achieve five levels of stimulation intensity—0, 0.19, 0.38, 0.56, and 0.74 Gy/s. The magnitude of the average decrease in bend frequency was found to increase with increasing X-ray dose rate [Figures 5F,H(5) = 38.2, *p* = 1.0×10^−7^]. *Post-hoc* tests found significant differences between the sham condition (0 Gy/s) and the 0.38 Gy/s (*U* = 333, Bonferroni corrected *p* = 0.008), 0.56 Gy/s (*U* = 376, Bonferroni corrected *p* = 3.7×10^−6^), and 0.74 Gy/s conditions (*U* = 340, Bonferroni corrected *p* = 6.9×10^−6^).

The paralysis assay was also repeated on the focused X-ray setup used for the avoidance experiments. For these experiments, crawling wild type and *pmyo-3::lite-1* nematodes were exposed to a 15 s pulse of 1 Gy/s focused X-ray stimulation. These experiments confirmed the finding that X-ray induced paralysis is observed in *pmyo-3::lite-1*, but not wild type worms. Additionally, the focused X-ray stimulation evoked egg ejection in five out of 10 *pmyo-3::lite-1* worms (Supplementary Video 7) and zero out of 10 wild type worms (Figures 5G,H).

## 5. Discussion

Here we have shown that wild type and LITE-1 intact *C. elegans* display a short-latency avoidance response to X-radiation that is absent in strains deficient for LITE-1. Additionally, transgenic expression of LITE-1 in *C. elegans* muscle cells was found to elicit a paralysis response to X-rays, demonstrating that transgenic expression of LITE-1 can confer X-ray sensitivity to otherwise X-ray insensitive cells. Together, these results suggest that LITE-1 can function as an X-ray sensitive receptor protein, playing a critical role in the transduction of X-ray signals into ionic currents and neural activity to produce behavioral responses.

We show using two different behavioral assays that X-rays produce behavioral responses in *C. elegans* that are dependent upon LITE-1. First, X-ray stimulation causes a dose-rate dependent increase in activity of wild type worms that is consistent with a locomotory avoidance response. The X-ray avoidance response was absent in worms with loss of function mutations in either LITE-1 alone or in both LITE-1 and GUR-3, but intact in worms with a loss of function mutation in GUR-3 alone. This indicates that the X-ray avoidance response depends upon LITE-1, but not GUR-3. Both LITE-1 and GUR-3 have previously been shown to be involved in the inhibition of pharyngeal pumping in wild type nematodes in response to UV (Bhatla and Horvitz, 2015), demonstrating that both are UV-sensitive photoreceptor proteins. However, only LITE-1 appears to be involved in the locomotory avoidance response to UV stimulation (Edwards et al., 2008; Bhatla and Horvitz, 2015), consistent with our observations in response to X-ray stimulation. Our next experiments show that the paralysis and egg ejection responses that have been reported in *pmyo-3::lite-1* worms in response to UV stimulation (Edwards et al., 2008; Bhatla and Horvitz, 2015; Gong et al., 2016) are similarly evoked by X-ray stimulation. This implies that X-rays, like UV, are capable of activating transgenically expressed LITE-1, leading to a calcium influx into muscle cells and muscle contraction (Gong et al., 2016). Together, our results demonstrate that LITE-1 can act as an X-ray sensitive receptor protein to mediate X-ray avoidance behavior in wild type nematodes and paralysis in *pmyo-3::lite-1* transgenic nematodes.

Our results with LITE-1 are the first demonstration of a protein being able to confer X-ray sensitivity in the form of a rapid behavioral response when trangenically expressed. Human intermediate-conductance Ca^2+^-activated K^+^ channels (hIK channels) have been found to confer X-ray sensitivity to otherwise X-ray insensitive HEK293 cells in the form of delayed (several minutes latency) and sustained voltage-dependent outward K^+^ currents after a 1 Gy dose of X-radiation (Roth et al., 2015). Thousands of studies have investigated the delayed biological effects of ionizing radiation, but only a few dozen have looked at immediate sensory detection of ionizing radiation. X-ray detection by ocular photoreceptor proteins such as rhodopsin has been suggested by reports of X-ray phosphenes (i.e., sensations of light produced by X-rays) experienced by astronauts and radiation-therapy patients (Lipetz, 1955; Fuglesang et al., 2006; Thariat et al., 2016). Additionally, there have been numerous reports of behavioral and electroretinogram (ERG) responses to X-rays and other types of ionizing radiation in diverse animal models (Baily and Noell, 1958; Bachofer and Wittry, 1961; Baldwin and Sutherland, 1965; Martinsen and Kimeldorf, 1972). The nearly ubiquitous finding that the retina must be in the scotopic, or dark-adapted state, in order to produce a response to X-radiation suggests that rhodopsin in rod cells, rather than cone opsins in cone cells, is involved in the response. Despite this, multiple researchers have reported that X-ray stimulation of the dark-adapted retina can increase its sensitivity to subsequent stimulation with visible light (Gaffey and Kelley, 1963; Dawson and Wiederwohl, 1965), suggesting that X-rays do not bleach the rhodopsin pigment, as does visible light. Additionally, it has not been investigated whether transgenic expression of rhodopsin outside of the specialized structure of the retina is capable of conferring X-ray sensitivity to different cell types. Although our experiments did not probe the X-ray sensitivity of GUR-3, other behaviors that are more dependent upon GUR-3 activation, such as the inhibition of feeding (Bhatla and Horvitz, 2015), might still demonstrate a behavioral effect of X-rays via GUR-3. Future studies could investigate whether other photoreceptors like GUR-3 become activated by X-radiation and confer X-ray sensitivity when transgenically expressed.

While sensory responses to X-rays have been documented in a variety of species, the cellular and molecular mechanisms by which X-radiation impinges on sensory system components have not been well explored. Similarly, the mechanisms by which X-ray activation of LITE-1 causes behavioral responses are not yet known. Given the similarity between UV and X-ray evoked behaviors, it is reasonable to assume that the cellular and molecular pathways downstream of LITE-1 activation are the same, regardless of which type of radiation is used to activate the receptor proteins. Because UV and X-ray photons exhibit major differences in their interactions with matter, however, the mechanisms by which the two types of radiation activate the photoreceptor protein may differ.

LITE-1 is unique among photoreceptor proteins, and its sequence does not contain any of the known chromophore binding sites found in nearly all other photoreceptor proteins that have been identified (Edwards et al., 2008). Despite lacking a small-molecule chromophore binding partner needed to capture photons for any other type of photoreceptor, isolated LITE-1 has been shown to absorb UV photons with astonishingly high efficiency, with measured extinction coefficients averaging two orders of magnitude larger than those of opsins (Gong et al., 2016). Interestingly, LITE-1’s high UV absorbance cross section is drastically diminished when particular residues of the protein, including two tryptophan moieties, are mutated (Gong et al., 2016). These mutations that inhibit photon absorption by LITE-1 also render the protein dysfunctional at mediating the UV paralysis response in *pmyo-3::lite-1* worms (Gong et al., 2016). Together, these data suggest that LITE-1, although unrelated to known photoreceptor families, may be a legitimate photoreceptor that can be activated by the direct absorption of UV photons *via* an intrinsic chromophore domain consisting of a particular arrangement of tryptophan residues.

In contrast, the chemical bonds and electron configuration that promote absorption of UV radiation by tryptophan have negligible effects on the absorption of X-radiation, which is primarily determined by atomic number (Bushberg et al., 2012). As a ~ 50 kDa protein, only about 1 out of 50 million molecules of LITE-1 can be expected to absorb an X-ray photon per Gy of irradiation (see Supplemental Information for calculations). In the present study, behavioral responses were observed in wild type nematodes within two seconds of the onset of X-ray stimulation at 1 Gy/s—i.e., at a total dose of <~2 Gy. Endogenous LITE-1 expression is low (Gong et al., 2016) and would not be expected to exceed 50 million functional LITE-1 proteins expressed at a given time in a single worm. It is improbable that activation of merely two LITE-1 receptors within a worm expressing 50 million LITE-1 receptors in total would be sufficient to drive a behavioral response. Considering further that this is a generous over-estimate, which is based on the highly improbable assumption that X-ray absorption by any atom within LITE-1 will yield the same result (i.e., LITE-1 activation), we reason that it is highly unlikely that the mechanism underlying LITE-1’s X-ray sensitivity involves direct X-ray photon absorption by the receptor, as has been demonstrated to be the case for UV photons. It is much more likely that secondary electrons and reactive oxygen species (ROS), which are generated in the hundreds to thousands for each X-ray absorption (Bushberg et al., 2012), are involved in the activation of LITE-1 by X-rays.

It has been shown that the predominant immediate effect of X-radiation on biological systems is the generation of ROS, primarily from the radiolysis of water (Zaider et al., 1988, see Supplemental Information). Interestingly, electron-rich tryptophan residues readily participate in redox reactions (Ehrenshaft et al., 2015). Tryptophan can become oxidized by UV radiation (Davies and Truscott, 2001; Pattison et al., 2012) and act as a photosensitizer to generate ROS upon UV photoabsorption (Davies, 2003; Chin et al., 2008). Furthermore, it has been shown that radiogenic hydroxyl radicals, hydrated electrons, and hydrogen atoms all react rapidly with tryptophan in aqueous solutions (Jayson et al., 1954; Armstrong and Swallow, 1969; Shen et al., 1987). As such, it could be that radiogenic ROS or their reaction products are interacting with the chromophoric tryptophan residues of LITE-1 in order to activate the receptor.

The precise mechanisms underlying the activation of LITE-1 are at present unknown, but ROS are heavily implicated in the process (Bhatla and Horvitz, 2015; Zhang et al., 2020; Ghosh et al., 2021; Quintin et al., 2022). When absorbed by endogenous photosensitizing molecules and moieties such as riboflavin and tryptophan, UV radiation generates ROS in biological specimens. Evidence suggests that these UV-generated ROS can mediate photoresponses in both *C. elegans* and yeast (Bhatla and Horvitz, 2015; Bodvard et al., 2017). GUR-3, which shares 40% homology with LITE-1, appears to function not as a true UV photoreceptor, but more like a chemoreceptor that detects hydrogen peroxide generated by UV (Bhatla and Horvitz, 2015). While LITE-1 does appear to be capable of functioning as a legitimate photoreceptor that absorbs UV photons (Gong et al., 2016), LITE-1 dependent behavioral and calcium responses to ROS have also been reported (Bhatla et al., 2015; Ghosh et al., 2021; Quintin et al., 2022). LITE-1 and GUR-3 mediated H_2_O_2_ detection in PHA and I2 neurons, respectively, has been found to rely on H_2_O_2_-reducing peroxiredoxin-2 (PRDX-2, Quintin et al., 2022), suggesting that the receptors may interact with an oxidized intermediate rather than directly with ROS. Interestingly, PRDX-2 deficiency had no effect on LITE-1 mediated photoresponses in PHA neurons (Quintin et al., 2022), possibly indicating divergent mechanisms for photon and ROS detection. Others have reported diminished phototaxis and ASH neuron photocurrents in the presence of H_2_O_2_, claiming that the ROS acts to inhibit, rather than activate, LITE-1 (Zhang et al., 2020). Still another study found that both light and ROS were required for LITE-1 dependent avoidance responses (Ghosh et al., 2021). While the activation of LITE-1 appears to be complex and uncertainty remains regarding the mechanism underlying LITE-1’s X-ray sensitivity, the present study has clearly demonstrated that X-rays effectively activate LITE-1.

For any neuromodulation technique, thermal effects of the deposited energy should be considered. *C. elegans* display robust thermotaxic behavior and are capable of detecting changes in temperature as small as 0.05°C (Clark et al., 2006). Despite the nematodes’ high thermal sensitivity and the relatively high X-ray dose rates employed in this study, X-ray stimulation did not lead to changes in worm activity in the *lite-1*(*ce314*) *X* and *gur-3*(*ok2245*) *X lite-1*(*ce314*) *X* mutant strains. These mutant strains are expected to have intact thermosensory capacities, as there is no evidence that LITE-1 plays any role in thermosensation. Moreover, even ignoring the rapid thermal diffusion and local heat dissipation, the largest cumulative X-ray dose delivered here (14.8 Gy or J/kg) would cause a temperature increase of only about 0.004°C (based on the heat capacity of water, 4.2 kJ/kg/K). As such, thermal effects of the X-ray stimulation protocol employed here can be considered negligible and dismissed as a potential concern.

Another potential limitation of the present study is the use of a single mutated allele of LITE-1—i.e., *lite-1*(*ce314*) *X*. It is possible that a background mutation in the *lite-1*(*ce314*) *X* strain could be responsible for the X-ray unresponsive behavioral phenotype; however, if this were the case it would not be expected that transgenically expressing LITE-1 in muscle cells would confer them with X-ray sensitivity. Our discovery that LITE-1 can confer X-ray sensitivity when transgenically expressed provides strong support for the hypothesis that the behavioral phenotype observed in the *lite-1*(*ce314*) *X* strain is in fact due to the *ce314* mutation in *lite-1*, rather than some unknown background mutation in the strain.

The baseline period of each experiment was collected with the X-ray unit on, but with a lead shutter blocking the X-ray beam. Given that the avoidance and paralysis responses are not seen until shortly after stimulation onset, when the shutter is opened, we can be confident that it is the X-rays, rather than some artifact of the X-ray unit, that is triggering the responses. Additionally, given that no responses are seen in the sham X-ray stimulation condition—in which the shutter opens and closes, but the X-ray tube current is set to 0 μA—we can be certain that the worms are not responding to the auditory signal of the X-ray shutter opening and closing.

It should be noted that this study employs relatively high X-ray dose rates, with significant responses seen above about 0.38 Gy/s. As a species, *C. elegans* have been found to exhibit a high resistance to ionizing radiation. For example, acute 𝛾 irradiation of young adult wild type worms has been found to only modestly decrease lifespan at doses over 1,000 Gy (Johnson and Hartman, 1988; Ishii and Suzuki, 1990). Nematode locomotion has been found to decrease after irradiation in a dose-dependent manner, with body bend frequency decreasing about 40% after a 541 Gy dose of 𝛾 rays (Sakashita et al., 2008). Even at the highest X-ray dose delivered in the present study (14.8 Gy), wild type worms displayed no significant decrease in locomotion, so the dramatic paralysis effect seen in the *pmyo-3::lite-1* strain is clearly in excess to any radiogenic dampening of locomotion observed at the doses employed here. Lower doses of ionizing radiation can, however, affect more radiosensitive processes such as ROS signaling, gene expression, and cellular replication. A 3 Gy dose was sufficient to induce changes in gene expression in stage L4 larva, which are generally more radiosensitive than adult nematodes (Nelson et al., 2002). Mammals are typically much more radiosensitive than nematodes, and routine human clinical radioscopy typically involves dose rates below 1 Gy/min. Future physiological investigations will reveal the appropriate X-ray pulse duration for LITE-1 activation, which likely occurs on a millisecond timescale not investigated by these initial behavioral studies. This and other important future experiments will be needed to optimize the efficacy of LITE-1 mediated neural modulation in order to minimize total exposure and off-target radiogenic effects.

In conclusion, we discovered that LITE-1 mediates an avoidance response to X-rays in wild type nematodes and paralysis and egg ejection responses to X-rays in *pmyo-3::lite-1* transgenic nematodes, providing strong evidence that LITE-1 can function as an X-ray sensitive receptor in *C. elegans*. The findings were robust and produced in two different labs with different X-ray units and experimental setups. This is the first study to identify an X-ray receptor protein that can be transgenically expressed in different cell types to acutely control the activity of those cells using X-rays. As such, it is the first demonstration of X-genetic control of cellular electrical activity in intact, behaving animals. Future studies will reveal whether LITE-1 also has the potential to enable minimally invasive X-genetic neural control in mammalian animal models.

## Data availability statement

The original contributions presented in the study are publicly available. This data can be found here: https://figshare.com/projects/WormX_Data/171786.

## Author contributions

KC, MR, PM, SF, JA, and MB contributed to conception and design of the study. KC, MR, and DG performed experiments. KC, MR, and AR analyzed data. KC wrote the first draft of the manuscript. KC, MR, and JA wrote sections of the manuscript. All authors contributed to manuscript revision, read, and approved the submitted version.

## Funding

This research was supported in part by an NSF EPSCoR Research Infrastructure grant to Clemson University [NSF 1632881] and by AlaEPSCoR GRSP15. The funders had no role in study design, data collection and analysis, decision to publish, or preparation of the manuscript.

## Conflict of interest

The authors declare that the research was conducted in the absence of any commercial or financial relationships that could be construed as a potential conflict of interest.

## Publisher’s note

All claims expressed in this article are solely those of the authors and do not necessarily represent those of their affiliated organizations, or those of the publisher, the editors and the reviewers. Any product that may be evaluated in this article, or claim that may be made by its manufacturer, is not guaranteed or endorsed by the publisher.
