# Supplementary material for: LITE-1 mediates behavioral responses to X-rays in Caenorhabditis elegans
Source: Front Neurosci. 2023 Aug 10;17:1210138. doi: 10.3389/fnins.2023.1210138 (PMC10450342; doi:10.3389/fnins.2023.1210138)
Supplement: Supplementary file 1 [file Data_Sheet_1.pdf]

Supplementary Material

LITE-1 Mediates Behavioral Responses to X-rays in *C. elegans*

Kelli E. Cannon*, Meenakshi Ranasinghe, Paul W. Millhouse, Ayona Roychowdhury, Lynn E. Dobrunz, Stephen H. Foulger, David M. Gauntt, Jeffrey N. Anker, and Mark Bolding*

*** Correspondence:** Corresponding Authors: kecannon@uab.edu, mbolding@uab.edu

1 Supplementary Data

Calculation of X-ray Absorption by LITE-1

When a 50 kDa LITE-1 molecule is exposed to 1 Gy of radiation, the expected value of energy absorbed directly from the radiation is approximately 5.3 x 10^{-4} eV, or approximately 2 x 10^{-8} 25 keV photons. In other words, approximately 1 in 5 x 10^{7} LITE-1 molecules will interact directly with an X-ray photon.

The average photon energy of a 50 kV unfiltered x-ray beam is approximately 25 keV. The mass-attenuation coefficient for the absorption of 25 keV photons by carbon is approximately 1 cm^2/g. The mass of a 50 kDa molecule is approximately (5 x 10^4) x (1.7 x 10^{-27} kg), or 8.5 x 10^{-23} kg (8.5 x 10^{-20} g), for a cross-section of 8.5 x 10^{-20} cm^2.

The effective atomic number of LITE-1 is approximately the same as that of water, so the energy absorbed from a beam of ionizing radiation by a LITE-1 molecule will be approximately the same as that absorbed by the same mass of water. The mass of a 50 kDa molecule is approximately 8.5 x 10^{-23} kg. Thus, the expected value of energy absorbed from 1 Gy of radiation is (1 J/kg) x (8.5 x 10^{-23} kg) = 8.5 x 10^{-23} J. 1 eV = 1.6 x 10^{-19} J, so the energy absorbed by the LITE-1 molecule is about (8.5 x 10^{-23} J) / (1.6 x 10^{-19} J/eV) = 5.31 x 10^{-4} eV.

The average photon energy of a 50 keV unfiltered X-ray beam is approximately 25 keV or 2.5 x 10^4 eV, so the expected number of photons absorbed by a LITE-1 molecule exposed to 1 Gy of radiation is approximately (5.31 x 10^{-4} eV) / (2.5 x 10^4 eV) = 2 x 10^{-8} X-ray photons.

Production of ROS

In pH-neutral aqueous solutions, X-rays produce approximately 230 nM solvated electrons, 222 nM hydroxyl radicals, 71 nM hydrogen peroxide, 60 nM hydrogen atoms, and 42 nM diatomic hydrogen per Gy of energy deposited (Spinks and Woods, 1990).

References

Spinks, J W T, and Woods, R J. (1990). An introduction to radiation chemistry. (3rd Edn.). New York: Wiley-Interscience.

2 Supplementary Figures and Tables

Supplementary Video Legends

Video 1. Wild type *C. elegans* exhibit a robust avoidance response to focused X-ray stimulation. The behavior of a crawling wild type adult hermaphrodite is shown before, during, and after 1 Gy/s X-ray stimulation. The location of the focused X-ray beam is indicated by the red circle. The nematode responds rapidly to the stimulation with an increase in forward locomotion to escape the X-ray beam.

Video 2. *Gur-3(ok2245) X* mutant *C. elegans* exhibit a robust avoidance response to focused X-ray stimulation. The behavior of a crawling *gur-3(ok2245) X* adult hermaphrodite, which has a loss of function mutation in GUR-3, is shown before, during, and after 1 Gy/s X-ray stimulation. The location of the focused X-ray beam is indicated by the red circle. The nematode responds rapidly to the stimulation with an increase in forward locomotion to escape the X-ray beam.

Video 3. *Lite-1(ce314) X* mutant *C. elegans* are deficient for an avoidance response to focused X-ray stimulation. The behavior of a crawling *lite-1(ce314) X* adult hermaphrodite, which has a loss of function mutation in LITE-1, is shown before, during, and after 1 Gy/s X-ray stimulation. The location of the focused X-ray beam is indicated by the red circle. The nematode does not display an overt response to the stimulation and remains in the path of the X-ray beam for the duration of the stimulus.

Video 4. *Gur-3(ok2245) X lite-1(ce314) X* mutant *C. elegans* are deficient for an avoidance response to focused X-ray stimulation. The behavior of a crawling *gur-3(ok2245) X lite-1(ce314) X* adult hermaphrodite, which has loss of function mutations in both GUR-3 and LITE-1, is shown before, during, and after 1 Gy/s X-ray stimulation. The location of the focused X-ray beam is indicated by the red circle. The nematode does not display an overt response to the stimulation and remains in the path of the X-ray beam for the duration of the stimulus.

Video 5. *Pmyo-3::lite-1* transgenic *C. elegans* exhibit a paralysis response to unfocused X-ray stimulation. The behavior of a swimming *pmyo-3::lite-1* adult hermaphrodite, which expresses transgenic LITE-1 in muscle cells, is shown before, during, and after 0.74 Gy/s X-ray stimulation. Unfocused X-ray stimulation was used to yield a diffuse, relatively even irradiation over the entire field of view. The nematode responds to the stimulation with muscle contraction and paralysis. Recovery of motor function occurs slowly after stimulation offset.

Video 6. Wild type *C. elegans* do not exhibit paralysis in response to unfocused X-ray stimulation. The behavior of a swimming wild type adult hermaphrodite is shown before, during, and after 0.74 Gy/s X-ray stimulation. Unfocused X-ray stimulation was used to yield a diffuse, relatively even irradiation over the entire field of view. The nematode does not display an overt response to the stimulation and no X-ray related muscle contraction or paralysis is observed.

Video 7. *Pmyo-3::lite-1* transgenic *C. elegans* exhibit paralysis and egg ejection in response to focused X-ray stimulation. The behavior of a crawling *pmyo-3::lite-1* adult hermaphrodite, which expresses transgenic LITE-1 in muscle cells, is shown before, during, and after 1 Gy/s X-ray stimulation. The nematode was targeted with focused X-ray stimulation and responds with muscle contraction, partial paralysis, and egg ejection.
